# Metabolic Modeling of Common *Escherichia coli* Strains in Human Gut Microbiome

**DOI:** 10.1155/2014/694967

**Published:** 2014-07-13

**Authors:** Yue-Dong Gao, Yuqi Zhao, Jingfei Huang

**Affiliations:** ^1^Kunming Biological Diversity Regional Center of Instruments, Kunming Institute of Zoology, Chinese Academy of Sciences, Kunming 650223, China; ^2^State Key Laboratory of Genetic Resources and Evolution, Kunming Institute of Zoology, Chinese Academy of Sciences, 32 Eastern Jiaochang Road, Kunming, Yunnan 650223, China; ^3^Kunming Institute of Zoology, Chinese University of Hongkong, Joint Research Center for Bio-Resources and Human Disease Mechanisms, Kunming 650223, China

## Abstract

The recent high-throughput sequencing has enabled the composition of *Escherichia coli* strains in the human microbial community to be profiled en masse. However, there are two challenges to address: (1) exploring the genetic differences between *E. coli* strains in human gut and (2) dynamic responses of *E. coli* to diverse stress conditions. As a result, we investigated the *E. coli* strains in human gut microbiome using deep sequencing data and reconstructed genome-wide metabolic networks for the three most common *E. coli* strains, including *E. coli* HS, UTI89, and CFT073. The metabolic models show obvious strain-specific characteristics, both in network contents and in behaviors. We predicted optimal biomass production for three models on four different carbon sources (acetate, ethanol, glucose, and succinate) and found that these stress-associated genes were involved in host-microbial interactions and increased in human obesity. Besides, it shows that the growth rates are similar among the models, but the flux distributions are different, even in *E. coli* core reactions. The correlations between human diabetes-associated metabolic reactions in the *E. coli* models were also predicted. The study provides a systems perspective on *E. coli* strains in human gut microbiome and will be helpful in integrating diverse data sources in the following study.

## 1. Introduction

*Escherichia coli* (*E. coli*) is the most widely studied prokaryotic model organism and an important species in the fields of biotechnology and microbiology.* E. coli* constitutes about 0.1% of human gut flora [1], which benefits human beings by providing supplemental nutrition, by enhancing nutrient acquisition, and by preventing the establishment of pathogenic bacteria within the intestine [2]. The study of this bacterium is both of importance for applications, such as environmental testing and metabolic engineering [3], and of interest as a fundamental physical problem. For example, a recent study demonstrated an obvious increase in the number of* E. coli* in the stool, while diarrhea was apparent [4].

In the recent five years, the flood of deep sequencing data has set the latest wave of microbiome research apart from earlier studies, with the ability to enumerate all of the cells in a complex microbial community at once [5]. For instance, using deep sequencing, the Human Microbiome Project (HMP) was launched to characterize the microbial communities found at several different sites on the human body and to analyze the role of these microbes in human health and disease [6, 7]. This switch from the low-throughput technique, culture-based enumeration, to the high-throughput technology of deep sequencing offers several advantages, including high accuracy, culture-free sampling, and comprehensive information. However, there are still two challenges to address. First, due to the huge data size and high complexity of the different algorithms, it is difficult to determine the exact roles of the various species in human microbiome, let alone strains of the same species. The composition of* E. coli* strains is of value to human health; for example, changes in the* E. coli* composition were observed associated with intestinal inflammatory disorders in human and mice [8, 9]. Second, most of the microbiota community structures obtained from sequencing were “static,” while the human microbiomes are diverse and dynamic. The diet changes, individual differences, sampling sites, and physical conditions are responsible for the dynamic responses of human microbiome [10–12]. However, the comprehensive responses of microbiome to the dynamic microenvironments can hardly be obtained from one or several samples.

To solve these problems, considerable efforts have been made to develop metabolic networks of* E. coli* [3, 13, 14]. These in silico models have been successfully applied in many fields. For example, they were frequently used in prediction of steady-state or dynamic responses of cells to changes in ecosystems [3]. In addition, the metabolic models can be easily integrated with other data sources, such as DNA sequencing [15], expression profiles [16], proteomics [17], or metabolomics [18]. Goals of such data integration efforts are (1) to gain a better understanding of the observable phenotypes of the cell, (2) to predict potential functions of molecular signatures, and (3) to apply these in silico models for biological discovery and engineering applications. As a result, integration of relevant omics data with metabolic models as a representative species in the human gut microbiota elucidates the changes in the gut microbiota.

In this study, we performed in silico modeling of metabolic networks of* E. coli* strains in human gut microbiome. First, we determined* E. coli* strains in human gut microbiome using 148 fecal metagenomes. Next, we reconstructed genome-wide metabolic network of common* E. coli* strains in human gut. Then, the cellular phenotypes were predicted and validated using the genome variation of* E. coli* and diet changes. The findings of the study will help in developing new technologies and tools for computational analysis and exploring the relationship between disease and changes in the human microbiome.

## 2. Materials and Methods

### 2.1. Human Gut Metagenomes and Reference Genomes

High-quality short reads of 148 human gut samples were retrieved from Human Microbiome Project (HMP, http://www.hmpdacc.org/). The sequenced and well-annotated* E. coli* genomes (totally 61 genomes) deposited in GenBank were downloaded from NCBI database (http://www.ncbi.nlm.nih.gov/), to build a reference genome database. The reads were aligned against the* E. coli* reference genome using BLASTN (version 2.2.27+) with *E* < 0.01, minimal 99% identity cutoff and considering the reads that were aligned onto only a single position in the reference genome.

### 2.2. De Novo Assembly and Identification of Genes

The reads of human gut samples were assembled by Newbler (454/Roche GS Mapper/Assembler), following the protocol in HMP [19]. The assembled scaffolds were aligned against* E. coli* genomes using BLASTN with minimal 99% identity cutoff and best hit output.

### 2.3. Reconstruction of Strain-Specific Metabolic Network

The* E. coli* pan-genome (the union of the gene sets of all the strains of a species) metabolic network has been generated in a recent study [20]. The strain-specific metabolic model could be reconstructed based on the pan-genome metabolic network. We generated metabolic networks for the common* E. coli* strains in human gut microbiome based on the pan-genome metabolic network.

In the process, we derived the strain-specific metabolic models using two commonly used algorithms of top-down metabolic reconstructions, including GIMME [21] and iMAT [22]. These two algorithms are different: the GIMME is a linear programming procedure, while the iMAT is a mixed integer linear programming procedure.

### 2.4. Predictions of Cellular Phenotypes Using Metabolic Network

Fluxes through reactions in the metabolic models can be predicted using flux balance analysis (FBA) [23]. In the process, fluxes are constrained by steady-state mass balances, enzyme capacities, and reaction directionality, which yield a solution space of possible flux values. Besides, FBA uses an objective function to identify flux distributions that maximize (or minimize) the physiologically relevant predicted solution. Cellular growth rate (biomass production in another word) was used as an objective function for FBA analyses performed in this study. The same biomass equation, growth (GAM) and nongrowth (NGAM) associated ATP requirement values, and PO (number of ATP molecules produced per pair of electrons donated to the electron transport system) ratio were used for all the* E. coli* models and were the same as that in iAF1260 model [24]. When the metabolic models were used to simulate the change of carbon source (e.g., from glucose to succinate), we obtained the corresponding optimal growth rates and flux distributions for all the reactions. If the uptake/secretion flux for a reaction in the optimal flux solution was reduced or increased by over 10% (flux.*x* > 1.1 × flux.*y* or flux.*x* < 0.9 × flux.*y*) between two conditions, we defined the reactions to be associated with the diet stress.

Uniform random sampling of the solution space for* E. coli* metabolic models in any environmental condition is a rapid and scalable way to characterize the structure of the allowed space of metabolic fluxes [25]. The set of flux distributions obtained from sampling can be interrogated further to answer a number of questions related to the metabolic network function. In the study, we studied how dependent two reactions within the* E. coli* network were on each other.

### 2.5. Flux Variability Analysis (FVA)

Biological systems often contain redundancies that contribute to their robustness. FVA can be used to examine these redundancies by calculating the full range of numerical values for each reaction flux in a network [26]. In FVA, the process is carried out by optimizing for a particular objective, while still satisfying the given constraints set on biological systems. In the study, FVA was applied to determine the ranges of fluxes that correspond to an optimal solution of the* E. coli* models determined through FBA. The maximum value of the objective function is first computed and this value is used with multiple optimizations to calculate the maximum and minimum flux values through each reaction.

## 3. Results

### 3.1. *E. coli* in Human Gut Microbiome

Deep metagenomic sequencing provides us the opportunity to explore the existence of a common set of* E. coli* species in human gut microbiome.

To obtain this goal, we built a nonredundant database of 61 sequenced and well-annotated* E. coli* genomes. After aligning the reads of each human gut microbial sample onto the reference database, we determined the proportion of the genomes covered by the reads (Methods). At a 99% identity threshold and 10-fold coverage (the genomes of* E. coli* strains are 5 M on average), we detected one in all gut samples, three in 80%, and seven in 60% of the 148 human gut samples (Table 1). We focused on the three common* E. coli* strains, including* E. coli* HS, UTI89, and CFT073. Other recent studies support our findings, including studies from human [27] and animal models [28].

Besides the genome-guided methods, the reads were used to perform de novo assembly, which can recover transcript fragments from regions missing in the genome assembly [29]. We first assembled metagenomes in 148 human gut microbiome samples using over 10 billion reads. Then, we mapped the 15 million gut scaffolds to the 293663 genes (target genes) of the 61* E. coli* genomes in the human gut. At a 99% identity threshold, over 60% of the target genes of the seven* E. coli* in Table 1 had at least 80% of their length covered by a single scaffold, indicating that the genes of these* E. coli* strains were significantly enriched in the gut scaffolds (Fisher's exact test, *P* < 10^−10^).

### 3.2. In Silico Metabolic Models of* E. coli* Strains

We generated genome-wide metabolic network of three common* E. coli* (*E. coli* HS, UTI89, and CFT073) from metabolic model of* E. coli* pan-genome using GIMME and iMAT algorithms.

**Figure 1:**
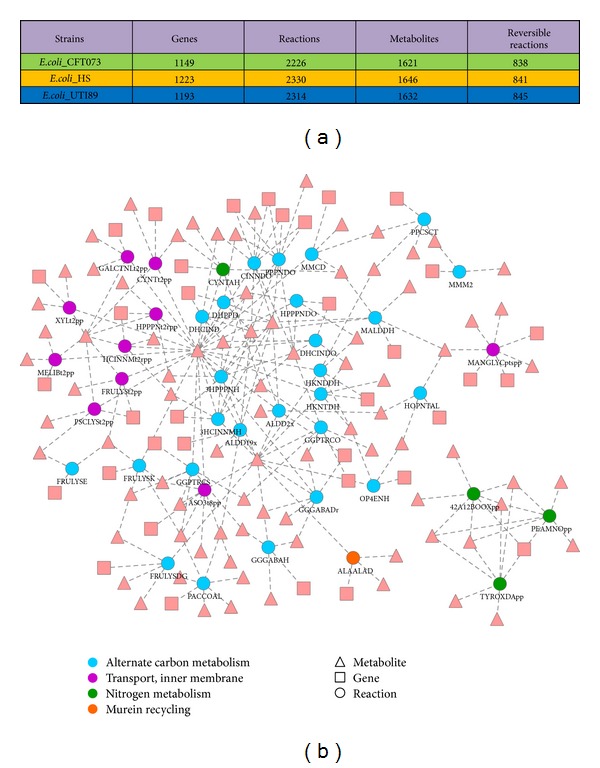
Comparisons of metabolic networks of three* E. coli* strains. (a) Basic parameters of metabolic models. (b) Strain-specific reactions in* E. coli* HS model.

The results indicate that the metabolic networks obtained with the two algorithms are identical (TEXT S1–S3 available online at http://dx.doi.org/10.1155/2014/694967). We then explored the differences in network properties among the three models. It shows that these models are different in network structure (Figure 1(a), Table S1). For example, compared with* E. coli* CFT073 and* E. coli* UTI89,* E. coli* HS model has 41 specific metabolic reactions catalyzed by 36 genes (Figure 1(b)). These reactions are associated with alternate carbon metabolism, murein recycling, nitrogen metabolism, and inner membrane transport. Most of the reactions tend to form a subnetwork rather than are scattered in an apparently random manner in the metabolic network. We also observed 32 different metabolites not included in all the three models (Table 2). Only three of the metabolites (including allantoate, tRNA-Ala, and tRNA-Phe) can be detected in the human metabolic model Recon2 [30], suggesting that most of these different metabolites are not involved in direct interactions of gut microbiome host. However, some of these metabolites are of importance to strain-specific characteristics and closely related to human-microbe interactions. For example, GDP-L-fucose plays important roles in microbial infection and numerous ontogenic events [31].

The genome-wide metabolic networks for* E. coli* CFT073 and UTI89 have recently been reconstructed based on the comparative genomics analysis [20]. We compared our models (TEXT S1–S3) with the previous ones and found that our models included more metabolic genes because the deep sequencing has been proven to lead to the identification of large populations of novel as well as missing transcripts that might reflect Hydra-specific evolutionary events [32].

### 3.3. Optimal Flux Distributions for* E. coli* Strains

**Figure 2:**
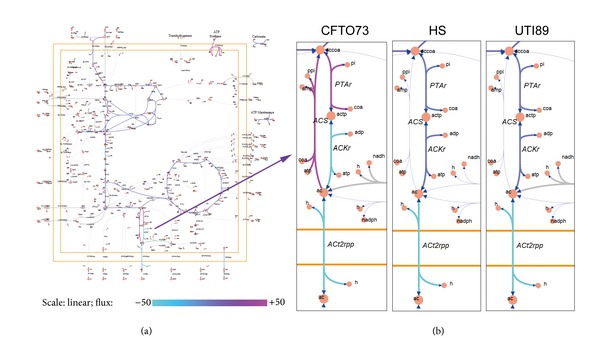
Flux balance analysis of metabolic models. The figure shows the core metabolic map (a) in* E. coli* and the reactions with different fluxes (b) among three* E. coli* models. ACS: acetyl-CoA synthetase; PTAr: phosphotransacetylase; ACKr: acetate kinase.

In the previous studies, one of the most fundamental genome-scale phenotypic calculations is the simulation of cellular growth using flux balance analysis (FBA) [25]. As a result, we defined biomass composition of the cell as the biomass objective function and performed FBA on the model in order to maximize the objective function. It shows that the optimal biomass flux for the three models are pretty close (optimal flux = 0.7287 for CFTO73, while optimal flux = 0.7367 for HS and UTI89). However, the optimal flux distributions are of different in the networks.  Figure 2 shows the optimal flux distribution map of core metabolic network in three* E. coli* strains. It shows that the fluxes of ACS (acetyl-CoA synthetase), PTAr (phosphotransacetylase), and ACKr (acetate kinase) in CFTO73 model are obviously different from that in the other two models.

We then estimated the effect of reducing flux through metabolic reactions on biomass production of three models. Two reactions ACOAD6F (acyl-CoA dehydrogenase, tetradecanoyl-CoA) and PGK (phosphoglycerate kinase) were taken as examples here (Figure S1). It shows that the growth rate is sustained near the optimal value over a range of values for PGK in all three models, indicating the same network robustness with respect to flux changes in the reaction (Figure S1A). However, the effects of reducing flux through ACOAD6F on growth are different between* E. coli* CFTO73 and the other two models (Figure S1B). Besides, the growth rate is sharply reduced after reaching the optimal value in HS and UTI89 models.

### 3.4. Dynamic Responses of Metabolic Networks to Changes in Carbon Sources

Although a few human gut microbiome projects have been launched, the interrelationships between our diets and the structure and operations of our gut microbial communities are poorly understood. Here, we predicted the human gut* E. coli*'s response to diet using metabolic modeling.

**Figure 3:**
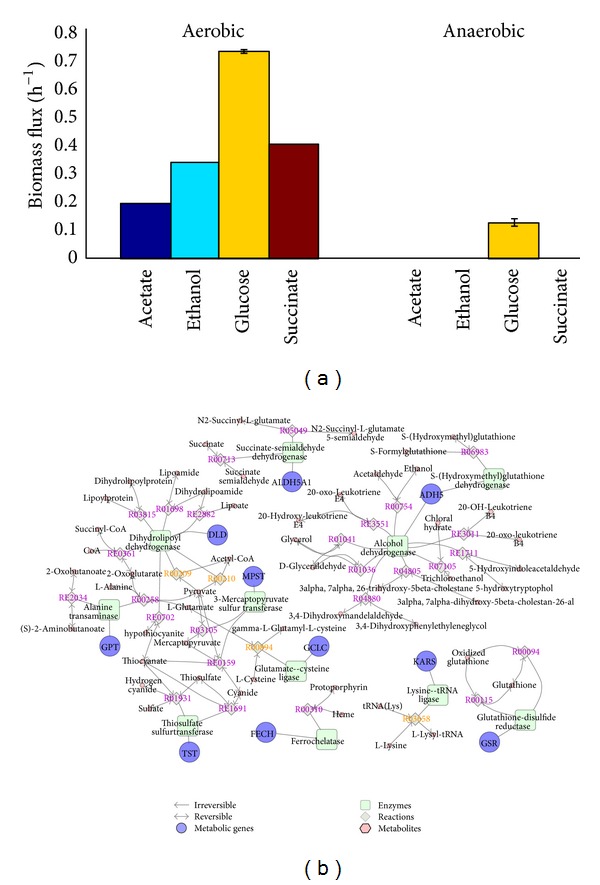
Optimal growth rates for* E. coli* strains on different carbon sources and the associated gene-protein reactions. (a) Optimal growth rates for* E. coli* strains on nutrition sources in human gut. The length of each bar represents the average optimal growth rates for three models on the same carbon source. (b) The diet stress-associated metabolic network in gut* E. coli*.

We simulated the optimal growth rates for three models on carbon source as acetate, ethanol, glucose, and succinate, respectively (uptake rate sets all changed to 9 mmol gDW^−1^ h^−1^). The average growth rates of three metabolic models corresponding to four diet conditions are shown in Figure 3(a). We can see that the growth rates for three models are similar in different conditions. Besides, it demonstrates substantially decreased anaerobic growth as compared with aerobic (18 mmol gDW^−1^ h^−1^) growth with the same glucose uptake rate, which was supported by recent studies that* E. coli* requires aerobic respiration to compete successfully in the mouse intestine [8, 9]. For* E. coli* strains in human gut, carbon sources are diverse, but glucose is most suitable for their growth.

These responses of* E. coli* to the diet changes involve many metabolic genes and pathways. We explored the perturbations in the metabolic networks and found 10 genes (including* ADH5*,* ALDH5A1*,* DLD*,* FECH*,* GCLC*,* GPT*,* GSR*,* KARS*,* MPST*, and* TST*) closely associated with the diet stress (Figure 3(b)). The glycolysis, gluconeogenesis, and glycerophospholipid metabolism were enriched in the metabolic reactions catalyzed by these genes (*P* < 10^−3^ using Fisher's exact test). Besides, we found that these enzymes were evolutionarily conserved from* E. coli* to human and were involved in the interactions between human and* E. coli* [14, 33]. Especially, nine out of these 10 genes (except* GPT*, glutamic-pyruvate transaminase) were found to be increased in human obesity [34].

### 3.5. Analyzing Flux Correlations in Diabetes-Associated Pathways in* E. coli* Using Sampling

Assessment and characterization of gut microbiota (*E. coli* acts as an integral component) has become a major research area in human type 2 diabetes, the most prevalent endocrine disease worldwide. A recent metagenomic research identified and validated over 400 type-2-diabetes-associated markers in* E. coli*, including over 100 metabolic genes [35]. In the study, we performed uniform random sampling for three models under glucose-limiting aerobic growth conditions to explore the relationships between the diabetes-associated pathways.

**Figure 4:**
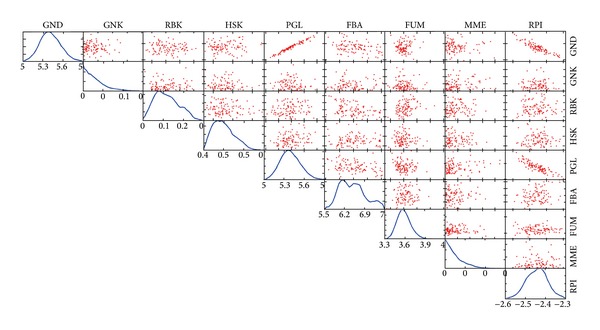
Flux sampling of* E. coli* HS model. Flux distribution histograms (diagonal) and pairwise scatterplots (off-diagonal) for diabetes-associated metabolic reactions in* E. coli* HS model. The* x*-axis of the histograms indicates the magnitude of the flux through the particular reaction. The scatterplots on the off-diagonal elements show the relationship between fluxes through two reactions. GND: phosphogluconate dehydrogenase; CAT: catalase; GNK: gluconokinase; RBK: ribokinase; HSK: homoserine kinase; TMK: thiamine kinase; PGL: 6-phosphogluconolactonase; FBA: fructose-bisphosphate aldolase; FUM: fumarase; MME: methylmalonyl-CoA epimerase; RPI: ribose-5-phosphate isomerase.

We detected 158 metabolic reactions in* E. coli* models that were associated with human type 2 diabetes (Table S2). It shows that these reactions participate in many subsystems, of which over 30% are associated with lipid metabolism and cofactor/prosthetic group biosynthesis. Correlation ships between some metabolic reactions can be observed in Figure 4. For example, PGL (6-phosphogluconolactonase) and GND (phosphogluconate dehydrogenase) fluxes are positively correlated in the* E. coli* HS model, whereas PGL shows negative correlation with RPI (ribose-5-phosphate isomerase) fluxes. The correlation ships between these diabetes-associated reaction fluxes are the same in other two models.

### 3.6. Flux Variability Analysis (FVA) of* E. coli* Models

FBA returns a single flux distribution that corresponds to maximal biomass production under given growth conditions. However, alternate optimal solutions may exist, which correspond to maximal growth. As a result, we performed FVA for the three* E. coli* models under glucose-limited aerobic growth conditions (glucose and oxygen were changed to 10 and 18 mmol gDW^−1^ h^−1^, resp.).

**Figure 5:**
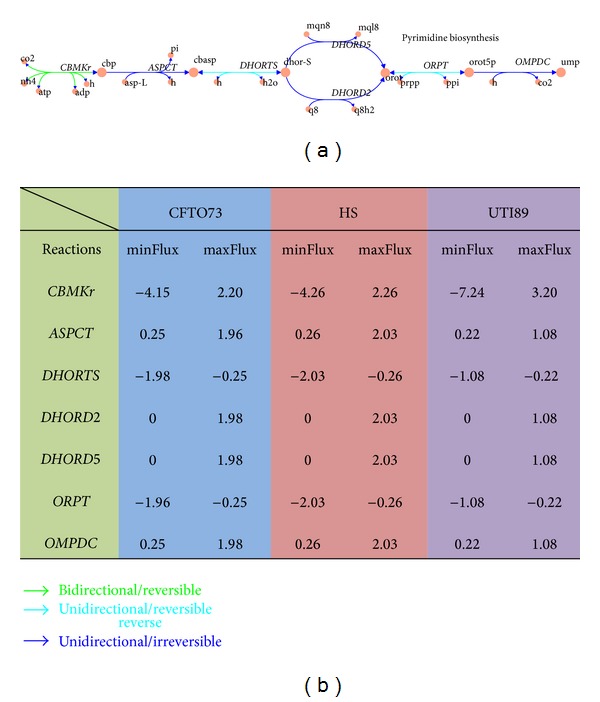
FVA of* E. coli* models. Shown is a map of metabolic reactions in pyrimidine biosynthesis pathway of* E. coli* models. Using FVA, the minimum (min) and maximum (max) allowable flux values for each reaction were determined. The values shown in the table correspond to the min and max allowable fluxes for each reaction shown in the map. The results were further characterized by the direction of predicted flux (bidirectional or unidirectional) computed using FVA. The full names of the metabolic reactions are included in TEXT S1–S3.

It shows that the minimum and maximum fluxes for the reactions in* E. coli* models are different.  Figure 5 illustrates FVA result for the seven reactions in pyrimidine biosynthesis. All the reactions have different flux range in three networks, especially carbamate kinase and dihydroorotic acid dehydrogenase.

## 4. Discussion and Conclusion

In our study, we determined the common* E. coli* strains in human gut microbial communities based on HMP datasets. We applied two widely used algorithms (GIMME and iMAT) to reconstruct genome-wide metabolic models for three common* E. coli* strains (*E. coli* HS, UTI89, and CFT073) and compared the network characteristics of these models. These models were then used to predict the cellular phenotypes and dynamic responses to the diverse gut microenvironment. The models were also applied in exploring the relationships between* E. coli* and human diabetes. The results will be helpful in exploring the dynamic responses of gut microbiome to the environmental perturbations.

The* E. coli* strains have been proven to be significantly different among individuals, although the species is abundant in human gut [36]. Although it is well accepted that the composition of* E. coli* strains in human gut flora is associated with health status, the exact molecular mechanism is still unclear. We detected the common* E. coli* strains in human gut and systematically compared their functions through in silico modeling, which have two advantages over the traditional methods. First, the sequencing data allows for a much more accurate determination of microbiome composition. The advent of next-generation sequencing (NGS) enabled several high-profile collaborative projects including the HMP Consortium (http://www.hmpdacc.org/project_catalog.html) and MetaHIT Consortium (http://www.sanger.ac.uk/resources/downloads/bacteria/metahit/), which have released a wide range of data on the human microbiome. Using these datasets, we applied different methods (genome-guided mapping and de novo assembly) to determine the common* E. coli* strains, making the following study of interconnectivity between gut microbiota, diet, and cell molecular responses available. Second, the metabolic modeling can allow us to see how a biological system might respond [37]. This will guide the wet lab experiments and avoid most of the mistakes in the process. In fact, developing computational methods capable of predicting metabolic flux by integrating these data sources with a metabolic network is a major challenge of systems biology [18]. For example, the predicted behaviors of diabetes-associated reactions in* E. coli* (Table S2) can be integrated with experimental validations to detect the causal genes in human diabetes.

The* E. coli* is regarded as the prototypical pluripotent pathogens capable of causing a wide variety of illnesses in a broad array of species, including pyelonephritis, diarrhea, dysentery, and the hemolytic-uremic syndrome [38]. In particlar, human gut* E. coli* and its relationship to complex diseases, such as cancer [39] and diabetes [40], has attracted increasing interest in the last few years. A question then arises: “How is it possible for this Jekyll and Hyde species to both coexist peacefully with its host and cause devastating illness?” [38]. The answer mainly lies in the existence of different strains of* E. coli* with variable pathogenic potential [41]. However, we can hardly draw a complete picture of how the* E. coli* strains respond physiologically to the complex gut microenvironment. Our study can provide valuable information based on the systematic comparisons of different* E. coli* strains. It shows that although the optimal growth rates are similar for three* E. coli* strains, the optimal flux distributions are different for three models, even in* E. coli* core reactions. The detected different reactions, such as ACS (acetyl-CoA synthetase) and PTAr (phosphotransacetylase) were approved to be involved in the virulence of* E. coli *and be associated with human complex diseases [35, 42]. The results can be integrated with other data sets, such as human clinical trials and virulence profiles, which will help establish the extent of commonality between food-source and human gut* E. coli *[43] and estimate the contribution of strain-specific reactions or genes to infections in humans.

We found that the* E. coli* responded distinctly to different gut diets and the stress-associated genes were closely associated with obesity. With the high prevalence of diet-induced health concerns, such as diabetes and obesity, there remains a need for approaches that treat the causal factors. Among these factors, gut microbiome is drawing more attention [35, 44] for it is suitable as disease markers and drug targets. For example, Qin et al. carried out a metagenome-wide association study which indicated that patients with type 2 diabetes have only moderate intestinal dysbiosis but that butyrate-producing bacteria are less abundant and opportunistic pathogens are more abundant in these individuals than in healthy controls [35]. The underlying mechanisms of interactions between gut microbiome and human health are complicated; however the stress-associated pathways (such as the detected the gluconeogenesis, and glycerophospholipid metabolism) may play important roles in the disease development. The diet changes first induced changes of involved metabolic genes (such as* ADH5*, alcohol dehydrogenase 5), which trigger the downstream signaling pathways. These signaling pathways mainly associated with immune responses and development [44, 45]. It is commonly accepted that the gut microbiota interacts with the immune system, providing signals to promote the maturation of immune cells and the normal development of immune functions [46]. The dynamic interactions between all components of the microbiota and host tissue over time will be crucial for building predictive models for diagnosis and treatment of diseases linked to imbalances in our microbiota.

In summary, the findings here represent a significantly expanded and comprehensive reconstruction of the* E. coli* metabolic network in human gut. This work will enable a wider spectrum of studies focused on microbe-host interactions and serve as a means of integrating other omics sets in systems biology.

## Supplementary Material

Figure S1: Robustness analysis of metabolic reactions in E. coli models.
Table S1: The reactions and metabolites in three E. coli metabolic networks.
Table S2: Metabolic reactions associated with human type 2 diabetes.
TEXT S1: Metabolic model for E. coli CFTO73 in SBML format.
TEXT S2: Metabolic model for E. coli HS in SBML format.
TEXT S3: Metabolic model for E. coli UTI89 in SBML format.

## Figures and Tables

**Table 1:** Common *Escherichia coli* strains in human gut.

*Escherichia coli* strains	Samples count^a^	Genome size	Gene counts	Protein count	Genes by *de novo* assembly
*E. coli* HS	148	4.6 M	4629	4377	3606
*E. coli* UTI89	134	5.0 M	5127	5017	3435
*E. coli* CFT073	125	5.2 M	5579	5369	3406
*E. coli* KO11FL	115	4.9 M	4756	4533	3512
*E. coli* NA114	94	5.0 M	4975	4873	3381
*E. coli* 536	90	4.9 M	4779	4619	3488
*E. coli* O127:H6 str. E2348/69	90	5.0 M	4890	4552	3179

^a^There are 148 individual samples in the analysis.

**Table 2:** Different metabolites in *E. coli* strains.

Metabolites	Descriptions	Formulas	Charges
4h2opntn	4-Hydroxy-2-oxopentanoate	C5H7O4	−1
acglc-D	6-Acetyl-D-glucose	C8H14O7	0
acmalt	Acetyl-maltose	C14H24O12	0
alatrna	L-Alanyl-tRNA(Ala)	C3H6NOR	1
all6p	D-Allose 6-phosphate	C6H11O9P	−2
alltt	Allantoate	C4H7N4O4	−1
allul6p	Allulose 6-phosphate	C6H11O9P	−2
cechddd	cis-3-(3-Carboxyethyl)-3,5-cyclohexadiene-1,2-diol	C9H11O4	−1
cenchddd	cis-3-(3-Carboxyethenyl)-3,5-cyclohexadiene-1,2-diol	C9H9O4	−1
cinnm	trans-Cinnamate	C9H7O2	−1
dhcinnm	2,3-Dihydroxicinnamic acid	C9H7O4	−1
dhpppn	3-(2,3-Dihydroxyphenyl)propanoate	C9H9O4	−1
dtdp4d6dm	dTDP-4-dehydro-6-deoxy-L-mannose	C16H22N2O15P2	−2
dtdprmn	dTDP-L-Rhamnose	C16H24N2O15P2	−2
frulysp	Fructoselysine phosphate	C12H24N2O10P	−1
gdpddman	GDP-4-Dehydro-6-deoxy-D-mannose	C16H21N5O15P2	−2
gdpfuc	GDP-L-Fucose	C16H23N5O15P2	−2
gdpofuc	GDP-4-oxo-L-Fucose	C16H21N5O15P2	−2
gg4abut	Gamma-glutamyl-gamma aminobutyric acid	C9H15O5N2	−1
ggbutal	Gamma-glutamyl-gamma-butyraldehyde	C9H16O4N2	0
ggptrc	Gamma-glutamyl-putrescine	C9H20O3N3	1
hkndd	2-Hydroxy-6-oxonona-2,4-diene-1,9-dioate	C9H8O6	−2
hkntd	2-Hydroxy-6-ketononatrienedioate	C9H6O6	−2
malt6p	Maltose 6′-phosphate	C12H21O14P	−2
man6pglyc	2(alpha-D-Mannosyl-6-phosphate)-D-glycerate	C9H14O12P	−3
op4en	2-Oxopent-4-enoate	C5H5O3	−1
pac	Phenylacetic acid	C8H7O2	−1
phaccoa	Phenylacetyl-CoA	C29H38N7O17P3S	−4
phetrna	L-Phenylalanyl-tRNA(Phe)	C9H10NOR	1
trnaala	tRNA(Ala)	R	0
trnaphe	tRNA(Phe)	R	0
urdglyc	(-)-Ureidoglycolate	C3H5N2O4	−1
